# Concentration-dependent Differences in Urinary Iodine Measurements Between Inductively Coupled Plasma Mass Spectrometry and the Sandell-Kolthoff Method

**DOI:** 10.1007/s12011-020-02381-8

**Published:** 2020-10-09

**Authors:** Yongze Li, Shuangning Ding, Cheng Han, Aihua Liu, Zhongyan Shan, Weiping Teng, Jinyuan Mao

**Affiliations:** 1 Department of Endocrinology and Metabolism and the Institute of Endocrinology, First Hospital of China Medical University, No.155 Nanjing Bei Street, Shenyang, 110001 Liaoning China; 2 Department of Molecular Pharmacology, Albert Einstein College of Medicine, 1300 Morris Park Avenue, Forchheimer 216, The Bronx, NY USA; 3 Department of Endocrinology and Metabolism, Peking University Third Hospital, Beijing, China

**Keywords:** Urinary iodine concentration, Inductively coupled plasma mass spectrometry, Sandell-Kolthoff reaction

## Abstract

A large amount of historical data regarding urinary iodine concentration (UIC) were measured with the Sandell-Kolthoff (S-K) method for iodine nutrition surveillance. The congruence in urinary iodine measurements between inductively coupled plasma mass spectrometry (ICP-MS) and the S-K method has been debated. A total of 2064 adult urine samples were included in the present study. The UIC measurement results obtained simultaneously by standardized ICP-MS and the S-K method were analyzed. The UIC obtained with ICP-MS was significantly higher than that obtained with the S-K method (158 μg/L vs. 148 μg/L, *p* < 0.001). The Bland-Altman difference plot showed a small but significant mean difference of 6.12 μg/L between the two methods. The stratified analysis showed that the correlation coefficient was higher in the UIC < 300 μg/L group than the UIC ≥ 300 μg/L group (0.93 vs. 0.88, *p* = 0.0001). The mean difference between the S-K and ICP-MS methods was positively correlated with the UIC. The ICP-MS and S-K methods were comparable when the UIC was less than 300 μg/L; however, UIC values between 300 and 600 μg/L should be compared with caution after considering the research objective. We do not suggest comparing UICs obtained from the ICP-MS and S-K methods in iodine monitoring studies if the UIC is greater than 600 μg/L.

**Electronic supplementary material:**

The online version of this article (10.1007/s12011-020-02381-8) contains supplementary material, which is available to authorized users.

## Introduction

Iodine is an essential component of thyroid hormones and normally obtained by the consumption of foods that contain iodine or iodized salt, and either low or high iodine intake may lead to thyroid disorders [1]. Iodine supplementation and monitoring programs have been implemented in most countries [2–4]. Urinary iodine concentration (UIC) reflects the dietary iodine intake and has been used as a marker for iodine status in population studies [5, 6].

Different techniques for the determination of urinary iodine, such as chemical methods and inductively coupled plasma mass spectrometry (ICP-MS), have been developed [7]. At present, UIC is commonly determined spectrophotometrically using the Sandell-Kolthoff reaction (S-K), or by ICP-MS [8]. The S-K method is cumbersome and time consuming, with significant chemical hazards such as arsenic and cerium. Additionally, as the S-K method relies on chemical kinetics for determination of iodine concentration, the assay is susceptible to form organic species which may chelate cerium and alter the reaction rate [9, 10]. The ICP-MS method is fast and accurate, offers precision that is equal to the S-K method, enables easier sample preparation, and has the potential for simultaneous multielement analysis [11].

At present, ICP-MS is considered the international standard for UIC; however, a considerable amount of historical data was acquired using the S-K method. For a valid comparison of modern data to results from historical studies, correlation studies must be performed for these two analytical methods [12].

Although the variation in iodine status of the population is not great and ranges from 50 to 300 μg/L, random urine iodine levels between individuals and within each individual vary greatly from 10 to 1000 μg/L [6]. The limitation of the S-K method is that the upper limit of the linear range of the standard curve is 300 μg/L; if the sample is above this value, it needs to be diluted and retested, and the process of dilution may affect the accuracy of the results [13]. A few reports have investigated whether the results of the two methods differ [11, 13–15]; however, the sample sizes of the studies were mostly less than 100 individuals, and the studies did not stratify for different levels of UICs.

Understanding the status of iodine nutrition among the population is important for designing proper interventions to reduce the iodine deficiency epidemic [6]. The objective of this study was to assess the consistency of the ICP-MS and S-K methods, especially at different levels of UICs, and to provide evidence for the future prevention and control of iodine nutrition on the basis of historical data.

## Materials and Methods

### Study Population

This study population was partially derived from the TIDE project (Thyroid disorders, Iodine Status and Diabetes: a National Epidemiological Survey) as previously described [16, 17]. A total of 2064 frozen urine samples from local residents were included in the study to cover a range of concentrations. The study was established in 2014 and approved by the Ethics Committee of China Medical University. The study adhered to the recommendations of the Declaration of Helsinki, and individuals aged more than 18 years who were not pregnant were included. All subjects provided written informed consent following a thorough explanation of the research procedures.

### Sandell-Kolthoff Measurements

The S-K method according to WS/T 107-2006 by ammonium persulfate digestion (As^3+^-Ce^4+^ catalytic spectrophotometry) was previously described (supplemental material) [18]. All chemicals were supplied by the Sinopharm Chemical Reagent Co., Ltd., China. All samples were measured using a UV-1600 spectrophotometer (Ruili Analytical Instrument Group Co., Ltd., China). A calibration curve was established with 0, 50, 100, 150, 200, 250, and 300 μg/L standard solutions. If the UIC of samples were ranged from 300 to 600 μg/L, the urine samples were double diluted by deionized water (1:1, 250 μL of sample plus 250 μL of deionized water); if they ranged from 600 to 1200 μg/L, they were quadruple diluted (1:3, 250 μL of sample plus 750 μL of deionized water), and then 250 μL diluted samples were digested and retested. The intra- and interassay coefficients of variation for the UICs were 3 to 4% and 4 to 6% at 66 μg/L and 2 to 5% and 3 to 6% at 230 μg/L, respectively.

### ICP-MS Measurement

UICs were determined using an Agilent® ICP mass spectrometer (Agilent 7700x, Agilent Technologies, USA). The collision mode with an octopole reaction system was used. The measurement method was described previously [11]. The diluent was composed of 1% tetramethylammonium hydroxide (Maya Reagent, Jiaxing, China), 0.01% Triton-X 100 (Sinopharm Chemical Reagent Co., Ltd., Shanghai, China), and 10 μg/L tellurium (Guobiao Testing & Certification Co., Ltd., Beijing, China) as an internal standard. The concentrations of the intermediate working calibrators were 0, 10, 25, 50, 100, 200, 300, 400, 800, and 1000 μg/L to establish a calibration curve. Urine samples and working calibrators were diluted by 1:10 (500 μL of sample/calibrator plus 4500 μL of diluent). The certified reference materials (GBW09108, GBW09109, and GBW09110) were purchased from Guobiao Testing & Certification Co., Ltd. (Beijing, China), and their target values were 70.8 μg/L, 143 μg/L, and 224 μg/L, respectively. The intraassay coefficients of variance were 2.3%, 1.4%, and 2.3% for GBW09108, GBW09109, and GBW09110, respectively, and the corresponding interassay coefficients of variance were 2.7%, 2.5%, and 2.4%, respectively.

### Statistical Methods

All statistical analyses were performed using SPSS Statistics for Windows version 22.0 (Chicago, IL, USA) and MedCalc Statistical Software version 15.6 (Ostend, Belgium), and *p* values at < 0.05 were considered significant. The chi-square test was used to test for differences between categorical variables. The data were tested for normality using the Kolmogorov-Smirnov test. The UICs obtained with either method were not normally distributed; therefore, the medians and interquartile ranges (IQRs) were reported, and Spearman correlation and nonparametric tests were performed. Passing-Bablok correlations and Bland-Altman difference plots were used to assess the agreement between results obtained by the ICP-MS and S-K methods. The samples were stratified by UIC as measured by the S-K method into < 300 μg/L, ≥ 300 μg/L, 300–600 μg/L, and > 600 μg/L groups since the urine samples were diluted if the UIC was higher than 300 μg/L. Severe iodine deficiency was defined as UIC < 20 μg/L, and iodine deficiency was defined as UIC < 100 μg/L [19].

## Results

Data were available for 2064 samples. The mean age of the adults was 45.73 years old (SD 14.65), and 38.67% were men. The prevalence of severe iodine deficiency labeled by the S-K and ICP-MS method was 0.48% and 0.53% (*X*^2^ = 0.048, *p* = 0.83), respectively. The prevalence of iodine deficiency labeled by the S-K method was significantly higher than those labeled by the ICP-MS method (27.62% vs. 24.81%, *p* = 0.04). Table 1 shows the UICs of the subjects as measured by the ICP-MS and the S-K methods. The UICs of the ICP-MS method ranged from 6 to 1124 μg/L (median: 158 μg/L; IQR: 101–245 μg/L). The corresponding values of the S-K method showed similar results, with UICs ranging from 5 to 1189 μg/L (median: 148 μg/L; IQR: 96–223 μg/L). The measurement values obtained with the ICP-MS method were significantly higher than those obtained with the S-K method (158 μg/L vs. 148 μg/L, *p* < 0.001).

**Table 1:** Comparison of UICs (μg/L) measured by the S-K and ICP-MS methods

Group	*N* (%)	ICP-MS Median (IQR)	S-K Median (IQR)	*p* value^a^
Total	2064 (100.00)	158 (101, 245)	148 (96, 223)	< 0.0001
Groups sorted by UIC using the S-K method				
<300 μg/L	1816 (87.98)	142 (94, 204)	135 (91, 191)	< 0.0001
≥300 μg/L	248 (12.02)	437 (370, 541)	456 (389, 585)	< 0.0001
300–600 μg/L	193 (9.35)	408 (349, 463)	424 (355, 479)	0.0004
> 600 μg/L	55 (2.66)	683 (587, 792)	768 (664, 866)	< 0.0001

^a^Comparison of UICs measured by the S-K and ICP-MS methods with the Wilcoxon test

When the UIC was < 300 μg/L, the UIC obtained by ICP-MS was significantly higher than that obtained by the S-K method (142 μg/L vs. 135 μg/L, *p* < 0.001). However, when the UIC was between 300 and 600 μg/L and greater than 600 μg/L, the UIC obtained by the ICP-MS method was significantly lower than that obtained by the S-K method (408 μg/L vs. 424 μg/L, *p* = 0.0004 and 683 μg/L vs. 768 μg/L, *p* < 0.0001, respectively).

**Fig. 1:**
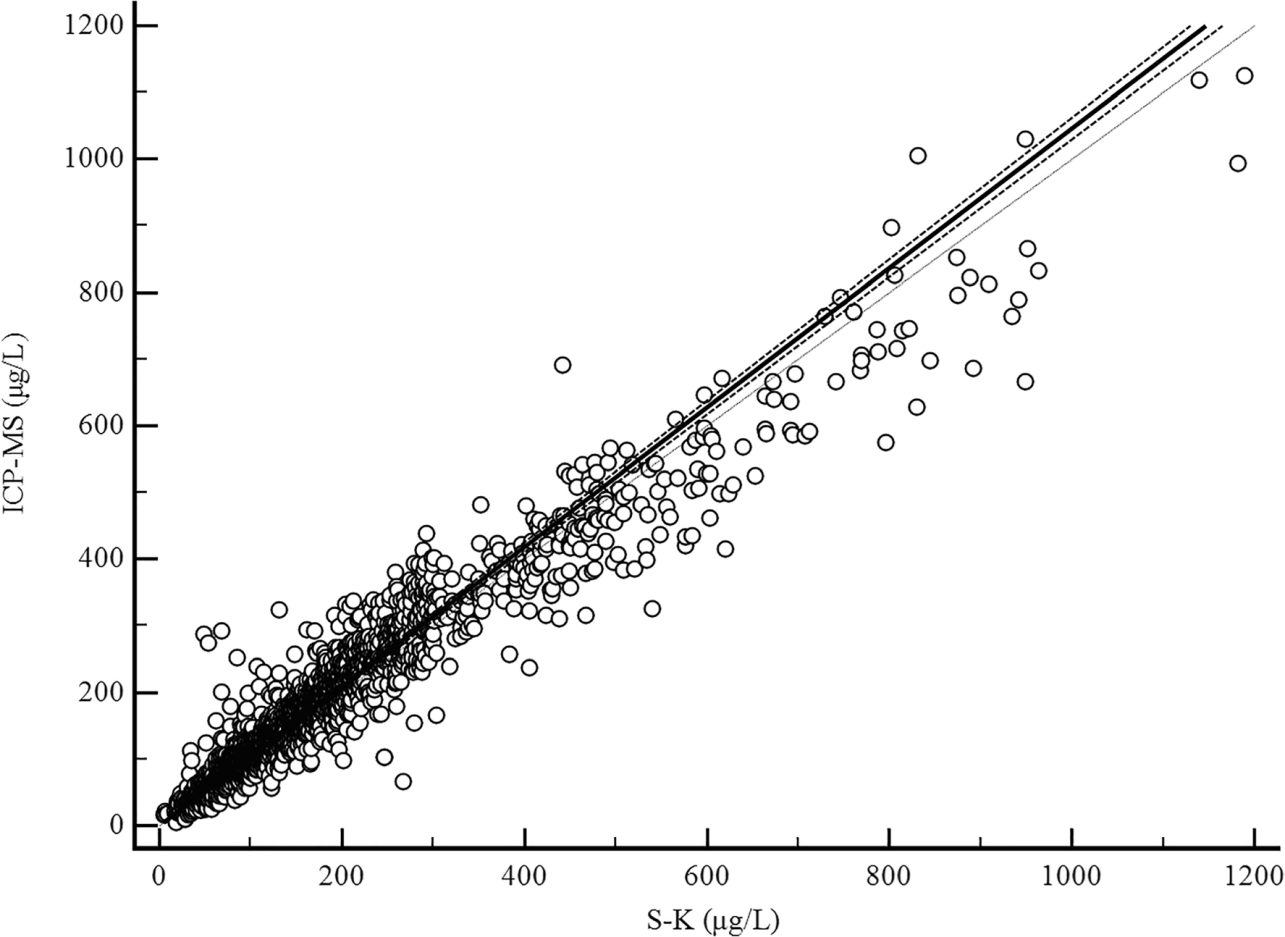
Comparison of the ICP-MS and S-K methods using the Passing-Bablok regression for the total population. The solid line represents regression line, the bold dashed line represents confidence interval, and the dotted line represents diagonal

The Passing-Bablok regression (Fig. 1) showed a very strong correlation between the two methods. The Passing-Bablok regression equation for both methods was y = 0.22 + 1.05x (x: S-K method; y: ICP-MS method). Constant differences were evaluated by calculating the intercept of the regression within the 95% CI (intercept: 0.22, 95% CI: − 1.89 to 1.98). The slope was 1.05 (95% CI: 1.03 to 1.06), and Spearman’s correlation coefficient was 0.95 (95% CI: 0.945–0.954, *p* < 0.001).

**Fig. 2:**
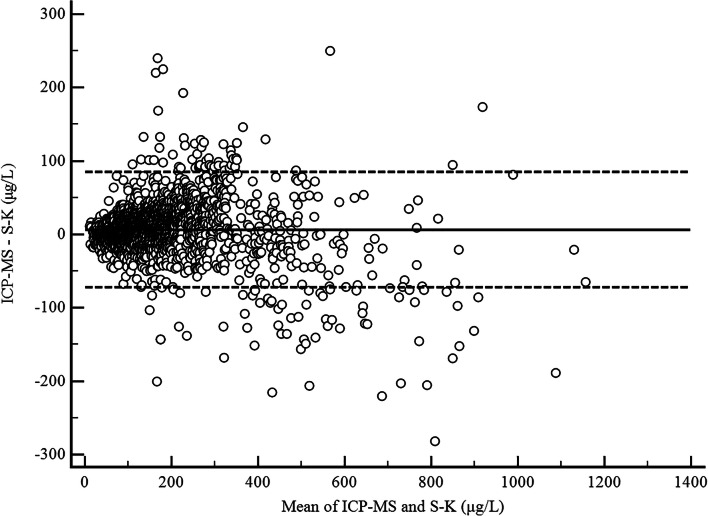
Comparison of the ICP-MS and S-K methods with the Bland-Altman plot for the total population. The solid line represents the mean difference, and the dashed line represents the 95% limit of agreement

The Bland-Altman plot (Fig. 2) for the UICs obtained by the ICP-MS and S-K method showed that the mean difference line was at 6.12 μg/L (95% CI: 4.40 to 7.85 μg/L). The differences between the individual results obtained by the two methods were concentration-dependent. The 95% limit of agreement (i.e., the interval defined by the mean differences ± 2 SDs) indicated that the variability in the difference between the ICP-MS and S-K results was between − 74.02 and 86.26 μg/L.

Stratified analyses of the Passing-Bablok regression and Bland-Altman plot were also conducted based on the UIC levels obtained by the S-K method (Table 2). For UIC < 300 μg/L obtained by the S-K method, Passing-Bablok regression showed a very high congruence between the two methods. The Passing-Bablok regression equation for both methods was y = − 8.95 + 1.14x (x: S-K method; y: ICP-MS method). Spearman’s correlation coefficient was 0.93, which was significantly higher than the corresponding value in the UIC ≥ 300 μg/L group (*p* < 0.0001) (Table 2). The differences between the individual results obtained by the two methods showed a small but significant mean difference of 10.85 μg/L (95% CI: 9.40 to 12.30 μg/L) between the two methods.

**Table 2:** Parameters of Passing-Bablok regression, Bland-Altman plot, and Spearman’s correlation

UIC of the S-K method	Passing-Bablok regression		Bland-Altman plot		Spearman’s correlation	
	Slope (95% CI)	Intercept (95% CI)	Mean difference (95% CI)	95% limits of agreement	Correlation coefficient (95% CI)	*p* value
< 300 μg/L	1.14 (1.12–1.15)	− 8.95 (− 11.05 to − 6.74)	10.85 (9.40–12.30)	− 52.07–73.77	0.93 (0.92–0.93)	< 0.0001
≥ 300 μg/L	0.89 (0.84–0.95)	31.55 (6.70–52.65)	− 28.49 (− 37.12 to − 19.85)	− 166.61–109.63	0.88 (0.85–0.91)	< 0.0001
300–600 μg/L	0.99 (0.90–1.10)	− 9.02 (− 52.90–31.03)	− 15.58 (− 23.92 to − 7.25)	− 133.00–101.84	0.78 (0.71–0.83)	< 0.0001
> 600 μg/L	1.10 (0.94–1.30)	− 154.71 (− 304.34 to − 25.30)	− 73.75 (− 96.18 to − 51.33)	− 239.65–92.15	0.84 (0.74–0.91)	< 0.0001

For the UICs ranging from 300 to 600 μg/L obtained by the S-K method, the Passing-Bablok regression showed a high congruence between the two methods. The Passing-Bablok regression equation for both methods was y = − 9.02 + 0.99x (x: S-K method; y: ICP-MS method). Spearman’s correlation coefficient was 0.78 (*p* < 0.0001), which was significantly lower than the corresponding value of 0.93 in the UIC < 300 μg/L group. The differences between the individual results obtained by the two methods showed a small but significant mean difference of − 15.58 μg/L (95% CI: − 23.92 to − 7.25 μg/L).

For the UICs > 600 μg/L values obtained by the S-K method, the Passing-Bablok regression showed a high congruence between the two methods. The Passing-Bablok regression equation for both methods was y = − 154.71 + 1.10x (x: S-K method; y: ICP-MS method). Spearman’s correlation coefficient was 0.84 (*p* < 0.0001). The differences between the individual results obtained by the two methods showed a significant mean difference of − 73.75 μg/L (95% CI: − 96.18 to − 51.33 μg/L).

## Discussion

Detection of urine iodine using the S-K method has been widely used in previous iodine nutrition monitoring; however, it requires tedious preparation of the sample before determination. The sensitivity, linearity, and reproducibility of the ICP-MS method have been proven sufficient to determine iodine concentrations in human plasma and urine [20, 21]. In our study, we observed a strong congruence between the ICP-MS and S-K methods in the whole population. The coefficients of the slope (test for slope = 1) differed significantly between the two methods, although the regression line was close to the bisectrix. A possible explanation was the concentration-dependent differences in the two methods, which were consistent with a previous study [11]. The Bland-Altman plot showed that the UIC measured by ICP-MS was 6.12 μg/L higher than that measured by the S-K method. That difference was acceptable in clinical examination; therefore, we considered that the S-K and ICP-MS methods showed good congruence and the test results of the two methods could be compared directly. The difference in the UIC < 300 μg/L results obtained by the S-K method compared with the ICP-MS method might be partly explained by the weak oxidation capacity of ammonium persulfate compared with perchloric acid because iodide needs to be rapidly and effectively oxidized to iodate to prevent the iodine from volatilization before photometric measurement through the S-K reaction [15].

In addition, we found that the UIC measured by ICP-MS was higher than that measured by the S-K method for UIC < 300 μg/L, which was different compared with previous studies [11, 14]. This finding might be due to a small sample size in the previous study that led to insufficient power to detect significant differences. In addition, each method considers a different set of parameters and has a slightly different standard operation procedure. However, the UIC obtained by the S-K method was higher than that obtained by ICP-MS for UIC ≥ 300 μg/L, which may be due to urine sample dilution for the S-K method. The possible explanations are as follows: urine samples contain iodine (which we are interested in) and sundry organics, proteins, amino acids (which we are not interested in and may interfere with the S-K reaction). Interfering species may slow the generation of the cerium chromophore, making a sample appear to have less iodine that it the true value. The samples of UIC ≥ 300 μg/L are double or quadruple diluted before digestion. As we dilute the iodine target in the samples, we are also diluting the interfering species that slow the S-K reaction, so the reaction speeds up and the measured value of iodine increases. The same samples tested by ICP-MS are diluted in a constant manner, not varying with sample concentration. In addition, we noticed that the greater the dilution the larger the deviation. When the UIC ranged from 300 to 600 μg/L, the mean difference between the two methods was 15.58 μg/L; and when UIC ≥ 600 μg/L, the mean difference reached 73.75 μg/L, which was unacceptable for clinical examination.

Differences in the UIC measurements may have been caused by a number of reasons, and the following factors may have played a relevant role. The urine matrix was diluted by water, and a previous study showed that test values obtained by the S-K method for high-UIC samples diluted by water were significantly higher than for low-UIC samples after dilution [22]. Another study showed that insufficient oxidants resulted in false high concentrations of pseudoiodine in urine due to interference with the incomplete ashed urine composition [23]. This variation could have implications for public health because monitoring for iodine-fortification programs usually focuses on the median UIC in the population. In the NHANES III conducted in 1988–1994, the median UIC of the population was 144.7 μg/L with the S-K method; however, in the NHANES 2001–2002, this value increased to 167.8 μg/L with the ICP-MS method [11]. The differences between the detection methods might have led to this increase to a certain extent, indicating the importance of accurate UIC measurements for adequate public health decisions. However, because the variations are more evident for individuals with excessive iodine intake and iodine fortification programs are more important for people with iodine deficiency, both methods could reliably quantify UICs.

This study is subject to several limitations. First, we could not analyze other potential influencing factors (such as drugs, diseases) that might have affected the results. Second, we did not verify whether the test value obtained by the S-K method for the high-UIC samples diluted by water was significantly higher than that for the low-UIC samples diluted by water, which would be relevant for explaining the observed differences in the UIC measurements. In addition, various digestion methods might lead to different results for the S-K method since we chose only ammonium persulfate as a digestion substance [15].

In conclusion, we demonstrated that the UIC results of the ICP-MS and S-K methods were comparable. In particular, the test results of the two methods could be compared directly when the UIC was less than 300 μg/L, which covered the normal adult UIC range. The results for UICs ranging between 300 and 600 μg/L should be compared only with caution after considering the research objective. However, we do not suggest comparing UICs from the ICP-MS and S-K methods in iodine monitoring studies if the UIC is greater than 600 μg/L. Further large-scale investigations with standard UIC measurements and adequate power should be conducted to generate a more precise estimate of the conversion formula between the ICP-MS and S-K methods for historical data comparisons.

## Electronic supplementary material

ESM 1 (DOCX 13 kb).
