# Supplementary material for: Concentration-dependent Differences in Urinary Iodine Measurements Between Inductively Coupled Plasma Mass Spectrometry and the Sandell-Kolthoff Method
Source: Biol Trace Elem Res. 2020 Oct 9;199(7):2489–95. doi: 10.1007/s12011-020-02381-8 (PMC8213661; doi:10.1007/s12011-020-02381-8)
Supplement: Supplementary file 1 — (DOCX 13 kb). [file 12011_2020_2381_MOESM1_ESM.docx]

The procedure of the S-K method according to WS/T 107-2006 involves the following steps:

1. Take 0.25ml of iodine standard series solution and urine sample respectively and put them in glass test tubes, add 1ml of ammonium persulfate solution to each tube, mix well, and heat each tube for 60 minutes in a heating block at 100℃. Cool the tubes to room temperature. The following analysis steps are performed in a stable temperature environment (room temperature or temperature control) between 20-35℃, and the temperature fluctuation is required to not exceed 0.3℃.

2. Add 2.5ml of arsenous acid solution to each tube, mix well, and leave aside for 15 minutes to allow the temperature to reach equilibrium; arrange the standard series of tubes in descending order of iodine concentration.

3. Add 0.3ml of cerium ammonium sulfate solution to each tube and quickly mix. A timer should be used to keep a constant interval (30 seconds), between additions to successive tubes.

4. When the absorbance value of the first tube (that is, the 300μg/L of iodine concentration tube in the standard series) reaches between 0.15-0.20, read its absorbency at 420nm in a spectrophotometer, and read successive tubes at the same interval (30 seconds) with 1cm colorimetric cup and water was used as a reference.

5. Result calculation (regression equation method):

The regression equation of the iodine concentration C (μg/L) of the standard curve and the absorbance value A is C=a+blnA (or lgA). The regression equation of the standard curve was calculated, and the absorbance value of the sample tube was substituted into the equation to calculate the iodine concentration in the measured sample.

6. Iodine concentration in urine: X=C*K

X-Urine iodine concentration, μg/L

C-The iodine concentration in the tested sample calculated from the regression equation, μg/L

K-dilution multiple of urine sample
